# EV-Encapsulated Mitochondrial miRNAs: Enhancing Cardiomyocyte Bioenergetics

**DOI:** 10.3390/ijms27052224

**Published:** 2026-02-26

**Authors:** Dhienda C. Shahannaz, Tadahisa Sugiura

**Affiliations:** 1 Digestive Disease & Surgery Institute, Cleveland Clinic, Cleveland, OH 44195, USA; dhiendaladdynasrul@gmail.com; 2 Department of Cardiothoracic and Vascular Surgery, Montefiore Medical Center, Albert Einstein College of Medicine, New York, NY 10467, USA

**Keywords:** extracellular vesicles (EVs), mitochondrial microRNAs, cardiomyocyte bioenergetics, regenerative cardiology, RNA therapeutics, non-cellular mitochondrial therapy, heart failure, systems biology, miRNA engineering, translational nanomedicine

## Abstract

Mitochondrial dysfunction lies at the core of numerous cardiac pathologies, yet restoring mitochondrial health remains a therapeutic frontier. In recent years, extracellular vesicles (EVs) have emerged as nature’s delivery nanocarriers, capable of transporting a wide array of biomolecules, including mitochondrial-associated microRNAs (mito-miRs). These miRNAs regulate bioenergetics, redox homeostasis, and apoptotic signaling—making them prime candidates for non-cellular mitochondrial therapy. This review explores the evolving landscape of mitochondrial miRNA encapsulation within EVs, focusing on their potential to restore mitochondrial transcriptional and metabolic programs governing ATP synthesis and redox balance, enhance cellular energy output, and mitigate oxidative stress. We integrate insights from stem cell biology, RNA epigenetics, systems cardiology, and bioengineering, offering a unifying framework for therapeutic applications across ischemic heart disease, heart failure, and chemotherapy-induced cardiomyopathy. An integrative narrative synthesis of recent peer-reviewed literature was performed across major biomedical databases, prioritizing mechanistic studies linking EV-mediated mito-miR delivery to cardiomyocyte mitochondrial function. By harmonizing multi-omic signaling, vesicle engineering, and mitochondrial medicine, this review seeks to guide future research toward targeted, customizable, and scalable bioenergetic interventions—unlocking a next-generation path for cardiovascular regeneration.

## 1. Introduction

The human heart is among the most metabolically demanding organs, consuming nearly 6 kg of ATP daily to sustain rhythmic contraction [1]. At the core of this immense energetic requirement lies the mitochondrion—not only as the powerhouse of cardiomyocytes but as a dynamic hub of calcium buffering, redox signaling, and apoptotic regulation [2]. In both inherited and acquired cardiac pathologies, mitochondrial dysfunction emerges as a key node of energetic collapse, initiating a cascade of maladaptive responses ranging from oxidative stress to electromechanical failure [2,3]. The failing heart, whether post-infarct or dilated, is typified by impaired oxidative phosphorylation (OXPHOS), fragmented mitochondrial morphology, and transcriptional silencing of energy-regulating genes—a bioenergetic bottleneck that directly limits the efficacy of regenerative therapies [2,3,4].

Induced pluripotent stem cell-derived cardiomyocytes (iPSC-CMs) provide a scalable and genetically tractable human cardiomyocyte model [4,5,6,7]. As a human-based platform, iPSC-CMs enable the interrogation of patient-specific genetic backgrounds, disease-causing variants, and pharmacological responses at scale, offering clear advantages over animal models in translational relevance and experimental throughput. Moreover, their capacity for expansion and differentiation supports high-content screening and mechanistic studies across diverse cardiac pathologies [4,5,6,7]. While traditional iPSC-CMs remain developmentally immature with respect to mitochondrial structure and function, recent metabolic and biophysical maturation strategies—such as PPARα agonist treatment, novel metabolic media formulations, and combined metabolic/mechanical conditioning—have advanced mitochondrial oxidative capacity, fatty acid utilization, and ATP production toward more adult-like profiles, though these cells still fall short of fully recapitulating adult cardiomyocyte energetics and excitation-contraction coupling in vitro [2,8,9,10,11].

However, their widespread clinical application is thwarted by their immature mitochondrial phenotype, which fails to recapitulate the robust respiratory capacity of adult myocardium [2,3,4,5]. This immaturity manifests as glycolytic preference, poorly developed cristae structure, and low mitochondrial DNA copy number—hallmarks that ultimately blunt their bioenergetic contribution in vivo [2].

Recent advances in extracellular vesicle (EV) engineering have opened new frontiers in organelle-targeted molecular delivery [12,13,14]. In this review, EV refers collectively to exosomes and microvesicles, excluding apoptotic bodies, unless otherwise specified. EVs, with their native lipid bilayer and inherent tropism, offer a minimally immunogenic, cell-free vehicle capable of transporting complex regulatory cargo, including microRNAs (miRNAs). Within this emerging space, mitochondrial miRNAs (mito-miRs)—including both mitochondrial genome-encoded miRNAs and nuclear-encoded miRNAs that localize to mitochondria—have garnered attention for their capacity to directly modulate mitochondrial gene expression, dynamics, and respiratory chain integrity [10]. Select mito-miRs, such as miR-181c, miR-378, and miR-210, have been shown to fine-tune key pathways in ATP generation, fission–fusion balance, and mitochondrial biogenesis [15].

In this review, as conceptual clarification, the term mitochondrial microRNAs (mito-miRs) is used as a functional umbrella encompassing two biologically distinct classes: (i) microRNAs encoded by the mitochondrial genome, and (ii) nuclear-encoded microRNAs that are imported into or functionally associated with mitochondria. While mitochondrial genome-encoded miRNAs remain comparatively rare and incompletely characterized, nuclear-encoded mitochondria-localized miRNAs constitute the dominant regulatory class in cardiomyocytes and mediate mitochondrial effects through post-transcriptional control of mitochondrial-encoded transcripts, nuclear-encoded mitochondrial genes, or mitonuclear signaling pathways. Unless explicitly stated otherwise, the term mito-miR in this manuscript refers to functionally mitochondria-associated miRNAs irrespective of genomic origin, with classification based on demonstrated bioenergetic impact rather than site of transcription.

Recent comprehensive reviews have catalogued mitochondrial and mitochondria-associated miRNAs across a wide spectrum of age-related and cardiovascular diseases, emphasizing their pleiotropic and context-dependent roles rather than disease exclusivity [16]. Notably, many canonical mito-miRs—including miR-210, miR-21, miR-378, and miR-181c—are recurrently observed across ischemic, metabolic, inflammatory, and degenerative contexts, underscoring that disease association alone is insufficient to infer dominant mitochondrial function.

Accordingly, this manuscript presents a comprehensive mechanistic review that integrates foundational and contemporary literature to contextualize mitochondrial bioenergetics and mitochondrial microRNA function in cardiomyocytes and advances the concept that precision extracellular vesicle-mediated delivery of mito-miRs may serve as a targeted strategy to enhance mitochondrial maturation. By recalibrating cardiomyocyte energetics at the molecular level, this framework addresses persistent limitations in iPSC-CM therapeutics and reframes metabolic regulation as a central axis of cardiac regeneration.

## 2. Methods

This review was conducted using an integrative, narrative-based analysis of peer-reviewed literature examining extracellular vesicles (EVs), mitochondrial-associated microRNAs (mito-miRs), and mitochondrial regulation in cardiomyocytes. A structured literature search was performed across PubMed, Scopus, and Web of Science to identify relevant studies published primarily within the past five years, with selective inclusion of earlier seminal works where mechanistic relevance or foundational significance justified inclusion.

Search terms were applied in various Boolean combinations and included: “extracellular vesicles,” “exosomes,” “mitochondrial microRNA,” “mito-miR,” “cardiomyocyte metabolism,” “mitochondrial dynamics,” and “cardiac ischemia.”

A total of 1142 records were identified (PubMed n = 412; Scopus n = 381; Web of Science n = 349). After removal of duplicates (n = 327), 815 records were screened based on titles and abstracts. Of these, 236 full-text articles were assessed for eligibility. The final qualitative synthesis included 104 studies that met predefined inclusion criteria; additional references were cited for background context, methodological standards, and mechanistic support.

### 2.1. Eligibility Criteria

Studies were included if they met the following criteria:

(i) Investigated EV biology, EV cargo loading, or EV-mediated molecular delivery;
(ii) Examined mitochondrial-associated miRNAs or miRNAs with demonstrated mitochondrial localization or function;
(iii) Evaluated downstream effects on mitochondrial bioenergetics, redox balance, apoptosis, mitochondrial dynamics, or structural integrity in cardiomyocytes or cardiac-relevant experimental models.

Exclusion criteria were applied to maintain mechanistic and contextual specificity. Studies were excluded if they

(i) Focused exclusively on non-cardiac tissues or disease models without mechanistic extrapolation to cardiomyocytes;
(ii) Examined miRNAs without evidence of mitochondrial association, targeting, or functional relevance;
(iii) Consisted of narrative reviews, commentaries, or editorials, which were excluded from synthesis tables but selectively cited where conceptual framing or contextual background was informative.

### 2.2. Data Evaluation and Synthesis

Eligible studies were evaluated qualitatively, with emphasis placed on mechanistic causality rather than outcome reporting alone. Both in vitro and in vivo preclinical studies were considered, encompassing stem cell-derived cardiomyocytes, primary cardiomyocytes, and relevant animal models of cardiac disease. Due to heterogeneity in EV sources, experimental platforms, delivery strategies, and outcome measures, no formal meta-analysis was performed.

Conceptual synthesis focused on identifying convergent mitochondrial signaling axes, temporal dynamics of intervention, and reported quantitative ranges of functional engagement across models. These findings were integrated into a translational framework highlighting bioenergetic thresholds, therapeutic timing, and engineering considerations relevant to EV-mediated mito-miR delivery in cardiac regeneration. This review adopts a comprehensive literature integration approach, synthesizing mechanistic, translational, and methodological studies relevant to mitochondrial bioenergetics and mitochondrial microRNA regulation in cardiomyocytes.

During the preparation of this manuscript, the authors used automated language-editing and overview functionalities available through publicly accessible tools (Google AI Overview) [16] to improve clarity and figure conceptualization. No machine-generated data, analyses, interpretations, or scientific conclusions were used in this work. All figures were manually developed and finalized by the authors to ensure rigorous scientific representation and conceptual accuracy.

## 3. Discussion

### 3.1. The EV Universe: Vesicular Biology and Cargo Versatility

Extracellular vesicles (EVs), once dismissed as cellular debris, have rapidly evolved into sophisticated mediators of intercellular communication. These nanoscale lipid bilayer particles—ranging from exosomes (30–150 nm) to microvesicles and apoptotic bodies [17]—are now recognized as bioactive vehicles with remarkable cargo specificity, structural resilience, and delivery potential [18]. In the context of cardiac regenerative medicine, EVs have become uniquely positioned as cell-free tools for the modulation of iPSC-CM phenotype, particularly in areas where direct cell transplantation faces immunologic, ethical, or maturation-related limitations [19,20].

At the molecular level, EVs exhibit a highly regulated cargo-loading process, influenced by endosomal sorting complexes (ESCRT), lipid raft domains, tetraspanins, and RNA-binding proteins such as hnRNPA2B1 and YBX1 [21]. This cargo includes functional proteins, lipids, long noncoding RNAs, and—critically—microRNAs (miRNAs), which retain the ability to reprogram target gene networks upon delivery. Recent innovations in EV engineering have enhanced this natural selectivity, enabling the encapsulation and delivery of custom therapeutic payloads with tissue or organelle-specific targeting sequences, including those directed toward mitochondria [22].

In the immature iPSC-CM, where native exosomal signaling may be insufficient to overcome metabolic inertia, the strategic delivery of exogenous EVs enriched in developmental or organelle-specific molecules offers a compelling workaround. Several studies have demonstrated that mesenchymal stem cell (MSC)-derived EVs or cardiac progenitor cell-derived EVs can partially rescue OXPHOS deficits in iPSC-CMs by modulating transcriptional and epigenetic landscapes [23]. Notably, this is not a one-way interaction—iPSC-CMs themselves generate EVs whose cargo content evolves with differentiation stage, stress response, and extracellular matrix cues, indicating that vesicular communication is not static but dynamically reflects cellular metabolic states [24,25].

As a delivery modality, EVs surpass many synthetic nanoparticles by virtue of their biocompatibility, low immunogenicity, and intrinsic ability to cross cellular and organellar membranes [26]. Moreover, their lipid composition mirrors that of their cell of origin, enabling efficient membrane fusion and intracellular routing [27]. In preclinical cardiac models, engineered EVs have shown the ability to home to injured myocardium, penetrate fibrotic tissue, and deliver miRNA cargo with temporal precision [28]—features highly attractive to immunologists designing tolerogenic therapies, stem cell researchers seeking maturity cues, and drug delivery specialists targeting metabolic syndromes.

The versatility of EVs as natural bio-shuttles introduces a paradigm shift: rather than simply mimicking healthy cells, we can now equip EVs to direct cellular fate, rewiring recipient cells at the metabolic, transcriptomic, and epigenetic levels. This capacity becomes especially potent when combined with mitochondrial-specific miRNAs (mito-miRs), a subset of regulatory RNAs capable of engaging the energetic core of cardiomyocytes [29]. As we will explore in the next section, these mito-miRs act as master regulators at the crossroads of redox biology, transcriptional rewiring, and mitochondrial maturation—offering a transformative route to bioenergetic optimization in iPSC-derived cardiac tissue.

### 3.2. Mitochondrial miRNAs: Gatekeepers of Bioenergetics

Among these regulators are mitochondrial microRNAs (mito-miRs)—used here as a functional term encompassing both mitochondrial genome-encoded miRNAs and nuclear-encoded miRNAs that localize to or regulate mitochondrial gene networks—whose bioenergetic effects extend across OXPHOS, redox balance, and mitochondrial dynamics. Importantly, EV-mediated delivery does not involve direct physical translocation of vesicles or miRNA cargo into mitochondria; instead, mitochondrial effects arise from post-transcriptional reprogramming of nuclear-encoded and mitochondrial-associated gene networks that govern bioenergetic output, redox balance, and organelle dynamics.

Cardiomyocyte function is inseparably tied to mitochondrial health [2], and at the core of this relationship lies a network of tightly regulated gene expression programs that govern ATP synthesis, redox balance, and organelle turnover [2]. Among the emerging regulators of these processes are mitochondrial microRNAs (mito-miRs)—a distinct subset of miRNAs either localized within the mitochondria or actively influencing mitochondrial gene networks through nuclear-encoded pathways. Their small size belies their regulatory complexity: mito-miRs operate as transcriptional and post-transcriptional modulators that fine-tune the expression of genes critical to OXPHOS, reactive oxygen species (ROS) detoxification, and mitochondrial biogenesis [15,30].

Given the arm-specific functionality of mature microRNAs, all miRNAs discussed herein are designated with their corresponding -5p or -3p strand where such information is available. This distinction is biologically consequential, as opposing arms of the same precursor miRNA can exhibit divergent target spectra, regulatory potency, and subcellular localization within cardiomyocytes [30,31,32,33,34]. In instances where original studies did not explicitly report arm specificity, the dominant functional strand identified in cardiomyocytes or cardiac-relevant models was adopted based on convergent evidence across independent datasets. This approach ensures mechanistic clarity while preserving translational relevance in the interpretation of mitochondrial miRNA activity [14,35,36,37].

Importantly, EVs do not physically enter mitochondria; rather, their cargo—including mitochondrial microRNAs—modulates mitochondrial gene networks indirectly by altering transcriptional and post-transcriptional programs governing ATP synthesis, redox balance, and organelle dynamics [13,22]. This mechanism enables functional reprogramming of mitochondrial output without direct organelle penetration, providing a non-cell-autonomous route to enhance bioenergetic capacity and oxidative homeostasis in cardiomyocytes [12,18].

In iPSC-CMs, where metabolic immaturity manifests as reliance on glycolysis and underdeveloped mitochondrial ultrastructure, mito-miRs present a promising route for reprogramming bioenergetic identity. For example, miR-181c has been shown to translocate into mitochondria and directly modulate COX1 expression, impacting electron transport chain efficiency. Similarly, miR-378 coordinates the PGC-1β/ERRγ axis, facilitating mitochondrial biogenesis and fatty acid oxidation (FAO)—both of which are essential for transitioning iPSC-CMs toward an adult-like metabolic state. Other mito-miRs, such as miR-210, play context-dependent roles in hypoxia adaptation by attenuating ROS generation while preserving mitochondrial membrane potential (Table 1) [36,37].

Recent reviews, including Kaur et al. [16], primarily organize mitochondrial microRNAs according to disease association, biomarker relevance, or cell death pathways. While this approach is valuable for mapping diagnostic prevalence and pathological overlap, it does not explicitly encode the directionality, magnitude, or mechanistic dominance of each miRNA’s impact on mitochondrial bioenergetics. Accordingly, Table 1 adopts a complementary framework that stratifies canonical mito-miRs by their dominant functional effects on ATP generation, redox balance, and mitochondrial structural integrity in cardiomyocytes.

Canonical mitochondrial and mitochondria-associated microRNAs (mito-miRs) are ranked using the Mito-miR Potency/Fidelity Index (MPI), a literature-derived composite metric developed in this review to qualitatively assess each miRNA’s net capacity to restore or impair cardiomyocyte mitochondrial function. This novel composite framework was constructed to normalize functional outcomes based on convergent evidence integrating reported effects on ATP production, ROS, mitochondrial membrane potential (ΔΨm), and apoptosis across cardiomyocyte-relevant models. It is not a clinical diagnostic tool, but rather a qualitative ranking system. The MPI integrates mechanistic findings reported over approximately three decades, incorporating functional inputs such as ATP production, pathological ROS, ΔΨm, electron transport chain (ETC) stability, mitochondrial dynamics, and apoptosis or mitophagy signaling in cardiomyocytes and cardiac-relevant experimental systems. Ranking is derived from consensus functional trends observed across in vitro and in vivo cardiomyocyte, cardiac injury, and metabolic remodeling studies, rather than from statistical aggregation or meta-analytic scoring. Individual parameters were evaluated qualitatively and conceptually normalized across studies to generate a relative hierarchy of bioenergetic influence.

Higher MPI rankings denote miRNAs with robust mitochondrial-stabilizing and ATP-preserving properties, whereas lower rankings identify miRNAs that impair mitochondrial maintenance or exacerbate energetic dysfunction and are therefore prioritized as candidates for inhibition rather than augmentation. When miRNA arm specificity (–5p or –3p) was not explicitly reported, the dominant functional arm documented in cardiomyocytes or cardiac-relevant models was used. Based on dominant bioenergetic behavior, mito-miRs occupy distinct positions along a bioenergetic control spectrum rather than constituting a uniform therapeutic class. This spectrum encompasses: (i) bioenergetic architects, which actively restore mitochondrial structure, oxidative metabolism, and ATP-generating capacity (e.g., miR-378, miR-499); (ii) contextual modulators, which prioritize cellular survival and redox buffering over energetic efficiency under stress conditions (e.g., miR-210, miR-21); and (iii) bioenergetic brakes or damage amplifiers, which accelerate mitochondrial dysfunction, apoptosis, or network fragmentation when overexpressed (e.g., miR-34a, miR-195, miR-15 family).

Importantly, disease context assignments in this table reflect dominant functional bioenergetic roles rather than diagnostic or associative classification. Accordingly, individual miRNAs may be linked to different cardiovascular disease entities across reviews depending on whether categorization is based on observational association, biomarker utility, or mechanistic effects on mitochondrial energetics, redox balance, and survival signaling. This functional framing supports precision extracellular vesicle (EV) cargo engineering, in which therapeutic benefit derives from selective miRNA augmentation or inhibition aligned with disease stage, metabolic demand, and mitochondrial stress context, rather than indiscriminate enrichment. Disease contexts listed here therefore reflect dominant bioenergetic relevance rather than diagnostic exclusivity; consistent with prior disease-association–based reviews, individual mito-miRs may appear across multiple cardiovascular and systemic pathologies depending on classification criteria [16].

#### Mechanistic Outcomes of EV-Delivered Mitochondrial miRNAs

Across cardiomyocyte models, delivery of mito-miRs via EVs consistently demonstrates three core mechanistic endpoints: (a). ATP ↑: Enhancement of OXPHOS and bioenergetic output through modulation of ETC complexes and upstream metabolic regulators (e.g., miR-378, miR-499). (b). ROS ↓: Attenuation of ROS via direct and indirect regulation of antioxidant enzymes, mitochondrial membrane potential stabilization, and redox-sensitive feedback loops (e.g., miR-210, miR-21). (c). Contractility preserved: Maintenance of cardiomyocyte contractile function through stabilization of mitochondrial dynamics, calcium handling, and ATP-dependent sarcomeric activity. These outcomes collectively position EV-encapsulated mito-miRs as tunable modulators of cardiomyocyte energetics, enabling targeted interventions that align with early injury windows and metabolic stress contexts, consistent with system-level predictions from integrative network modeling and translational multi-omic analyses.

The influence of mito-miRs extends beyond metabolic flux. Integrative transcriptomic analyses have revealed that several mito-miRs also target nuclear-encoded genes involved in mitophagy, calcium homeostasis, and the unfolded protein response, highlighting their position at the intersection of metabolic programming and cellular stress resilience [45,53,61]. Importantly, many of these miRNAs act in a redox-sensitive manner, forming feedback loops wherein oxidative stress induces changes in miRNA expression, which in turn remodel mitochondrial performance [49,58]. Such dynamic regulation offers a level of control well-suited to applications requiring adaptive metabolic responses, including cardiac repair and tissue engineering [22,50,51].

Beyond direct regulation of metabolic flux, several mitochondrial and mitochondria-associated miRNAs operate within tightly coupled redox feedback circuits that link oxidative stress sensing to transcriptional and post-transcriptional control. miR-21 (Table 1), through repression of PDCD4 and PTEN, attenuates stress-induced apoptotic signaling while indirectly stabilizing mitochondrial redox balance, particularly in ischemia–reperfusion contexts. In parallel, miR-146a (Table 1) [45,46] functions as an immunometabolic modulator by targeting TRAF6 and IRAK1, thereby dampening NF-κB-driven inflammatory cascades that secondarily amplify mitochondrial ROS production. Conversely, hypoxia-inducible miR-210 exemplifies a context-adaptive redox regulator: by suppressing ISCU and select electron transport chain components, miR-210 transiently constrains mitochondrial respiration, limiting electron leak and oxidative damage under low-oxygen conditions. Collectively, these miRNAs form self-regulating redox loops in which mitochondrial stress both shapes and is reshaped by miRNA expression, positioning mito-miRs as dynamic regulators of oxidative homeostasis rather than static metabolic switches [62].

**Figure 1:**
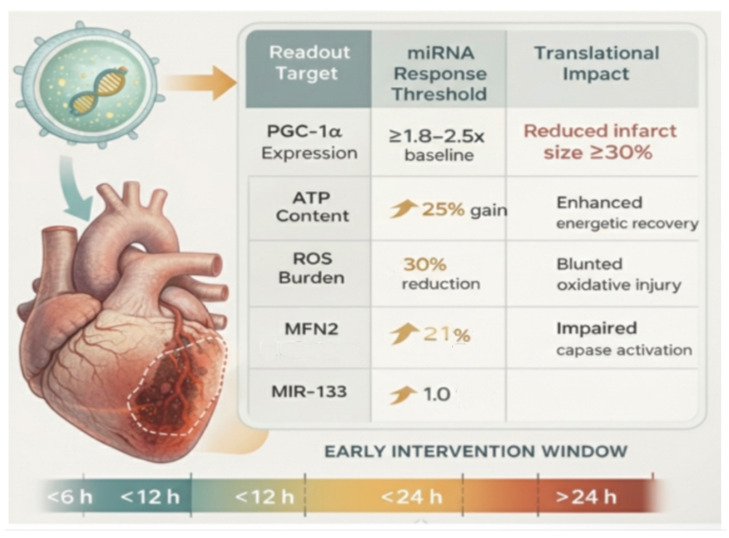
Proposed Translational Thresholds for EV-Delivered Mito-miR Therapy.

This schematic Figure 1 summarizes quantitative and temporal bioenergetic engagement thresholds inferred from convergent preclinical myocardial ischemia–reperfusion and mitochondrial gain-of-function studies that are required for extracellular vesicle (EV)-mediated mito-miR delivery to achieve meaningful cardioprotection. Across murine and large-animal models, reductions in infarct size of approximately 30% [12,62] are consistently associated with preservation of myocardial ATP content by ~20–30%, attenuation of ROS burden by 25–40%, and stabilization of mitochondrial membrane potential prior to widespread permeability transition pore opening. Notably, a decline in ATP of ~2030% has been linked to irreversible cardiomyocyte death, underscoring the importance of early bioenergetic rescue [63].

Within this framework, mito-miR-driven modulation of mitochondrial transcriptional regulators is proposed to operate within defined amplitude ranges. Moderate induction of the PGC-1α axis (approximately 1.8–2.5-fold) is sufficient to amplify downstream FAO and respiratory chain gene expression while avoiding maladaptive uncoupling observed at supraphysiologic levels [64,65]. Parallel engagement thresholds are illustrated for complementary mitochondrial pathways, including CPT1B-mediated FAO (≈1.5–2.0-fold), antioxidant reinforcement via MnSOD [66] and NRF2 signaling to achieve ≥30% ROS reduction, and modest upregulation of mitochondrial fusion regulators such as MFN2 (≈1.3–1.8-fold) to preserve network integrity [67] and suppress apoptosis [68]. Arrow ↑ means increase in response threshold. 

Critically, the timing of these molecular effects is constrained to an early intervention window, optimally within 12 h and no later than 24 h following ischemic insult, before caspase activation and irreversible cardiomyocyte loss dominate tissue fate, such as miR-133 levels which often halved after a heart attack and a 1-fold restoration is a standard therapeutic target [69]. Together, these parameters define a translational evaluation framework in which EV-delivered mito-miRs are assessed not solely by cellular uptake, but by their capacity to reach defined mitochondrial bioenergetic thresholds within a narrow temporal window. This positions EV-based miRNA delivery as a quantitatively tunable strategy for mitochondrial rescue rather than a passive molecular supplement [68]. In contrast to disease-association-based frameworks such as that proposed by Kaur et al. [16], which catalog mitochondrial miRNAs according to pathological context or cell death signaling, Table 2 applies a therapeutic design logic. Here, miRNAs are assigned based on dominant mitochondrial failure modes and directionally appropriate modulation (augmentation versus inhibition) required to restore cardiomyocyte bioenergetics.

### 3.3. Mitochondrial Maturation in Cardiomyocytes: Genetic and miRNA Regulation

Mitochondrial maturation in cardiomyocytes encompasses a coordinated program of structural, metabolic, and transcriptional remodeling that enables the developmental transition from glycolysis-dominant energy production toward FAO-driven OXPHOS and high-capacity ATP synthesis. This process is characterized by progressive densification of mitochondrial cristae, expansion of mitochondrial DNA (mtDNA) copy number, a substrate preference shift from glucose toward fatty acids, and activation of the PGC-1α/ERRγ/TFAM transcriptional axis governing mitochondrial biogenesis and respiratory competence [1,2,3,5,8,9,10,93]. Recent high-resolution metabolic, ultrastructural, and systems-level analyses have demonstrated that these features are tightly coupled rather than independently acquired, such that cristae architecture, electron transport chain assembly, and substrate utilization mature in concert to support excitation–contraction coupling and sustained cardiac workload [10,79,80].

In contrast, iPSC-CMs consistently exhibit delayed or incomplete mitochondrial maturation, manifested by sparse cristae organization, reduced mtDNA content, attenuated FAO capacity, and persistent reliance on glycolytic metabolism, collectively limiting their fidelity in modeling adult myocardial energetics and stress responses [2,3,5,8,9,10,11]. Although recent advances integrating metabolic conditioning, biophysical cues, and electrical stimulation have markedly improved iPSC-CM maturation, these approaches converge on a shared mechanistic endpoint: restoration of mitochondrial transcriptional and metabolic programs governing ATP synthesis and redox balance [8,9,10]. Notably, accumulating evidence indicates that this maturation trajectory is not governed solely by nuclear transcription factors, but is increasingly modulated by mitochondrial and mitochondria-associated microRNAs that fine-tune oxidative metabolism, mitonuclear communication, and stress adaptability [12,13,14,15,28,53].

Among these, mito-miRs such as miR-378 and miR-499 have emerged as key regulators of cardiomyocyte mitochondrial maturation, acting upstream of metabolic gene networks to reinforce FAO dominance, stabilize respiratory chain function, and support cristae integrity during developmental and regenerative transitions [14,15,28,53]. Conversely, stress-responsive mito-miRs including miR-21, miR-210, and members of the miR-15 family preferentially promote metabolic downscaling, redox buffering, or apoptotic priming under hypoxic or pathological conditions, reflecting a context-dependent trade-off between energetic efficiency and cell survival [15,39,40,45,55]. These observations position mitochondrial microRNAs as critical post-transcriptional regulators that bridge mitochondrial maturation and injury adaptation, providing a mechanistic rationale for EV-mediated mito-miR delivery strategies aimed at selectively reinforcing bioenergetic maturation rather than indiscriminate metabolic activation. In this framework, effective cardiac regeneration depends not on uniform mitochondrial stimulation, but on precise modulation of mito-miR networks aligned with developmental stage, metabolic demand, and mitochondrial stress state.

### 3.4. EV-Encapsulation Mechanisms: Selectivity, Engineering, and Tropism

The utility of extracellular vesicles (EVs) as delivery vehicles for therapeutic microRNAs—particularly those targeting mitochondrial networks—hinges on the specificity, efficiency, and fidelity of cargo encapsulation [94]. Native EV biogenesis follows endosomal or plasma membrane budding pathways, wherein molecular selectivity is driven by post-transcriptional RNA modifications [95], RNA-binding protein (RBP) affinity [96], and membrane lipid microdomains [97,98,99]. Yet, this intrinsic selectivity, while biologically robust, is not inherently optimized for therapeutic precision. To overcome these limitations, bioengineering strategies have emerged to control miRNA cargo loading, enhance vesicle targeting, and direct subcellular localization—redefining EVs as programmable nanocarriers.

Among the best-characterized mechanisms of selective RNA packaging are those mediated by RBPs such as hnRNPA2B1, AGO2, YBX1, and SYNCRIP [94], which recognize specific sequence motifs or structural elements in miRNAs to facilitate sorting into EVs. Advances in synthetic biology have further enabled the design of artificial scaffolds—aptamer-like structures [100] or fusion proteins—that tether therapeutic miRNAs to vesicle-enriched RBPs [101], dramatically increasing loading efficiency. In parallel, modular engineering of donor cells using lentiviral [102,103] or CRISPR-based systems [104] allows stable overexpression of desired miRNAs alongside targeting motifs, ensuring both sustained production and consistent packaging of mitochondrial miRNA cargo.

Tropism, the ability of EVs to home to specific cell types or subcellular compartments, can be modulated through surface display technologies. Genetic fusion of vesicle membrane proteins (e.g., Lamp2b, CD63, or tetraspanins) [105] with targeting peptides—such as cardiomyocyte-specific motifs or mitochondrial localization sequences—has demonstrated enhanced delivery accuracy in vitro and in vivo. Moreover, lipid modification strategies, including the insertion of synthetic ligands [106] or pH-responsive moieties [107], offer an additional layer of targeting refinement for navigating complex tissue environments, such as fibrotic myocardium or ischemic zones.

Importantly, the interplay between vesicle structure, cargo identity, and extracellular milieu governs biodistribution and uptake. Bioinformatic modeling of EV pharmacokinetics and vesicle-cell interaction networks has begun to inform rational design of delivery systems optimized for disease-specific contexts. These developments collectively position EVs as modular, adaptable platforms for intracellular targeting—including mitochondrial pathways—with a level of control previously unattainable using conventional delivery vehicles.

**Figure 2:**
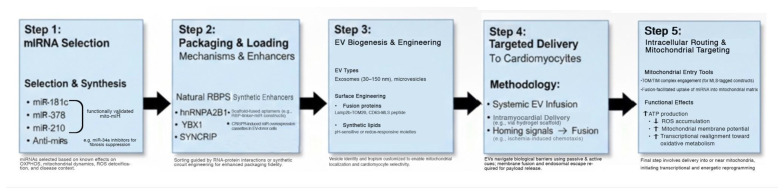
Modular Pathway for Engineering EV-Encapsulated Mitochondrial miRNAs for Targeted Cardiomyocyte Bioenergetic Reprogramming. Schematic representation of the modular engineering pipeline for extracellular vesicle (EV)-encapsulated mitochondrial microRNAs (mito-miRs) [12,13,108]. This multilayered logic map illustrates the progressive stages of therapeutic design, from miRNA candidate selection [28,29] and RNA-binding protein (RBP)-mediated packaging, through vesicle engineering and targeting, to intracellular mitochondrial delivery. Each layer incorporates both natural and synthetic biological components to enhance precision, selectivity, and functional bioenergetic impact in iPSC-derived cardiomyocytes [2,10,13]. In Step 2, hnRNPA2B1 acts to recognize GGAG motifs, and YBX1-SYNCRIP [20,95] acts as sequence- or structure-guided binding. Scaffold-fused aptamers example is RBP-linker-miR constructs [21,104]. The platform offers a scalable, tunable, and organelle-specific delivery strategy for RNA-based bioenergetic therapy, see [12,13,108,109,110] for examples of organelle-specific EV targeting. Arrows depict sequential directionality: The chronological order of experimental stages, where the output of one phase (e.g., loaded extracellular vesicles) serves as the essential input for the next, from Step 1 through Step 5.

As these encapsulation strategies mature, their translational relevance becomes increasingly evident in cardiac pathologies marked by metabolic insufficiency and mitochondrial dysfunction. The application of EV-delivered mitochondrial miRNAs in clinically relevant conditions such as ischemic heart disease, heart failure with preserved ejection fraction (HFpEF), chemotherapy-induced cardiotoxicity, and viral myocarditis forms the basis of the following section (Figure 2).

While EVs naturally exhibit tissue tropism, mitochondrial or cardiomyocyte-specific delivery can be enhanced via mitochondrial localization sequences (MLS), cardiomyocyte-targeting peptides, or surface-modified ligands. These strategies have been reported in preclinical studies [12,13,108,109,110], and allow EV cargo, including mito-miRs, to preferentially engage mitochondrial networks without requiring direct organelle entry. For full mechanistic details and applied examples, readers are referred to previous studies from our group and others [12,13].

**Figure 3:**
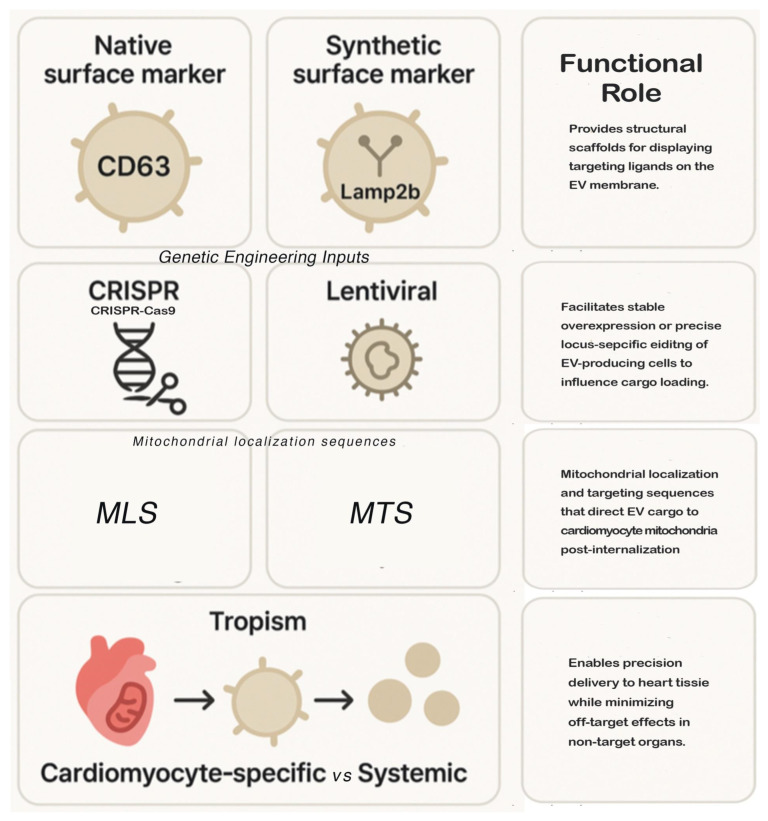
Engineering Landscape of EV surface Modification and Tropism. Schematic representation of the genetic and biochemical inputs used to program extracellular vesicle (EV) identity [12,13]. Surface markers (CD63, Lamp2b) [104,107] provide scaffolds for ligand display, while genetic tools like CRISPR and Lentiviral vectors [21,36,101] enable stable modification of parent cells. Intracellular routing is controlled via mitochondrial localization/targeting sequences (MLS/MTS) to ensure cargo reaches the mitochondrial matrix [12,13,108,109], collectively enabling a transition from systemic distribution to cardiomyocyte-specific tropism [17,20,25].

Figure 3 summarizes the modular engineering strategies used to control extracellular vesicle (EV) surface identity, intracellular routing, and tissue tropism, highlighting the design principles that enable precision delivery of mitochondrial microRNAs (mito-miRs) to cardiomyocytes. At the foundational level, EVs inherit native membrane proteins from their parent cells, among which tetraspanins such as CD63, CD81, and CD9 serve as canonical exosomal markers and scaffolds for further functionalization. These proteins not only define EV biogenesis pathways but also provide accessible anchoring points for engineered ligands or targeting peptides without compromising vesicle integrity.

Lamp2b [107,108,109,110,111], an endosomal–lysosomal membrane protein enriched on exosomal membranes, is frequently exploited as a fusion backbone for displaying targeting motifs on the EV surface. Genetic fusion of Lamp2b with cardiomyocyte-binding peptides, homing ligands, or organelle-specific signals enables directional uptake while preserving endogenous membrane composition. Such strategies allow EVs to transition from passive carriers to programmable delivery platforms.

Upstream of surface modification, EV-producing cells can be genetically engineered using lentiviral transduction or CRISPR-based genome editing [111,112]. Lentiviral systems enable stable overexpression of targeting constructs or cargo-sorting proteins, offering high efficiency and scalability, albeit with insertional considerations [113,114]. In contrast, CRISPR-mediated knock-in or regulatory editing allows precise, locus-specific modification of EV-associated genes, reducing variability in cargo loading and improving batch-to-batch reproducibility. These approaches directly influence EV yield, composition, and targeting fidelity.

Beyond tissue targeting, intracellular and organelle-specific delivery is achieved through incorporation of mitochondrial localization signals (MLS) or mitochondrial targeting sequences (MTS) [115,116] which facilitate post-uptake trafficking of EV cargo toward cardiomyocyte mitochondria. When combined with RNA-binding adaptors, these signals enhance the probability that delivered mito-miRs engage mitochondrial gene networks rather than remaining confined to the cytosol.

The net outcome of these engineering layers is controlled tropism, ranging from cardiomyocyte-specific delivery—critical for minimizing off-target effects—to systemic distribution when broader immunometabolic modulation is desired.

### 3.5. Cardiac Applications of Mitochondrial miRNAs via EVs

Mitochondrial dysfunction is a shared pathological hallmark across a wide spectrum of cardiac diseases [79,86,117]. Despite divergent etiologies, conditions such as ischemic heart disease (IHD), heart failure with preserved ejection fraction (HFpEF), anthracycline-induced cardiotoxicity, and viral myocarditis converge upon perturbations in OXPHOS, mitochondrial dynamics, and redox homeostasis [12,118,119]. These molecular disruptions are often accompanied by shifts in transcriptomic and proteomic profiles that drive maladaptive remodeling [90]. Within this context, extracellular vesicle (EV)-mediated delivery of mitochondrial miRNAs (mito-miRs) offers a promising avenue for disease-specific metabolic correction and cardioprotection [12,58].

**Figure 4:**
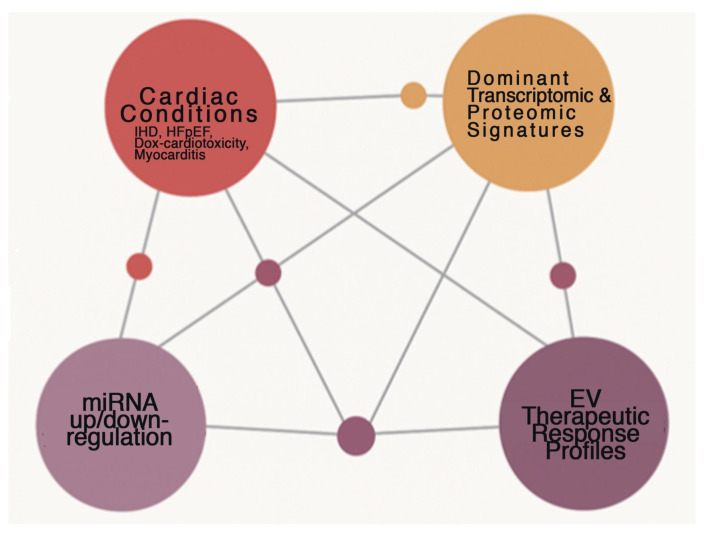
Disease-Omics Map Overlaying Mito-miRs in Cardiac Conditions. The integration of multi-omics data reveals that cardiac pathology is not merely a result of protein dysfunction but a systemic failure of regulatory networks. As shown in Figure 4, there is a dense interconnection between clinical Cardiac Conditions and miRNA regulation. Specifically, Mito-miRs serve as critical nodes that influence the Dominant Transcriptomic & Proteomic Signatures of the failing heart. The map illustrates that EV Therapeutic Response Profiles are directly linked to these miRNA shifts. This suggests that by monitoring the miRNA cargo within EVs, clinicians can gain a real-time ‘read-out’ of the heart’s proteomic state and its subsequent response to therapy. The intermediate nodes represent specific molecular targets where mitochondrial dysfunction meets systemic signaling, providing a roadmap for precision medicine in treating IHD and myocarditis. Integration nodes between lines represent the intersection or “crosstalk” points where two data types overlap. For example, a node between Cardiac Conditions and miRNA regulation represents the specific miRNAs known to be dysregulated in a particular disease. Connecting lines denote a statistically significant correlation or a known biological pathway connecting the two data domains. The density of the network suggests that none of these favors operate in isolation; rather, a change in a cardiac condition directly influences the transcriptomic signature and the miRNA profile simultaneously.

In IHD, prolonged ischemia and reperfusion injury result in extensive mitochondrial membrane depolarization, cytochrome c leakage, and ATP depletion [68,113]. Preclinical studies demonstrate that delivery of EVs enriched with mito-miRs such as miR-210 and miR-21 mitigates reperfusion-induced oxidative stress by downregulating ROS-generating enzymes and restoring mitochondrial membrane potential [39,43]. These interventions further activate pro-survival signaling pathways, including PI3K/Akt and NRF2, providing cytoprotection without the need for cell engraftment (Figure 4) [18,22].

In HFpEF, a syndrome characterized by diastolic dysfunction, fibrosis, and impaired mitochondrial substrate utilization, the therapeutic window remains narrow due to patient heterogeneity and absence of effective metabolic modulators [78]. EVs carrying miR-181c and miR-378 have been shown to enhance FAO and mitochondrial biogenesis in stiffened myocardium, improving lusitropic function and cellular energy profiles [52,115,116]. This suggests a role for mito-miR therapies not only in reversing energetic insufficiency but in altering the trajectory of disease progression.

Anthracycline (e.g., doxorubicin)-induced cardiotoxicity is directly linked to mitochondrial DNA damage, topoisomerase IIβ interference, and ROS accumulation [82]. EV-based delivery of mito-miRs that stabilize mitochondrial DNA repair pathways or buffer redox overload—such as miR-23a or miR-499—has demonstrated efficacy in preserving cardiac mitochondrial integrity and attenuating apoptosis in both in vitro and murine models [36,115,116,117,118]. Notably, this approach offers a non-interfering, cardioprotective adjunct to chemotherapeutic regimens.

In viral myocarditis, particularly that caused by coxsackievirus or SARS-CoV-2, mitochondrial dysfunction is exacerbated by immunometabolic derangement and direct viral protease targeting of mitochondrial fusion proteins [85]. Preliminary investigations indicate that EVs bearing immunoregulatory mito-miRs may rebalance innate immune activation while restoring mitochondrial network architecture—representing a dual-action therapeutic paradigm.

Collectively, these findings highlight the potential of mito-miR-loaded EVs to address both structural and energetic deficits in cardiac pathology. Integration into precision cardiology is increasingly guided by high-throughput multi-omic datasets, including single-cell transcriptomics, epitranscriptomic modifications, and proteomic landscapes, which refine mitochondrial-targeted interventions [77,118].

#### 3.5.1. Stratification of Mitochondrial miRNAs by Clinical Context

Based on convergent mechanistic and preclinical evidence, mitochondrial miRNAs can be rationally stratified according to preventive, therapeutic, and disease-modifying applications rather than treated as a uniform class. The functional impact of EV-delivered mito-miRs can be conceptually stratified into preventive, therapeutic, and diagnostic contexts, reflecting both the temporal dynamics of cardiac injury and the underlying mechanistic axis of each miRNA.

1. miR-210: Predominantly active in acute ischemic events, where hypoxia-driven ROS accumulation and transient ETC suppression necessitate rapid redox adaptation. Its transient delivery via EVs mitigates oxidative injury and preserves mitochondrial membrane potential, functioning as an early-intervention, context-specific therapeutic [53,119].
2. miR-378: Optimally deployed in chronic heart failure, where metabolic stiffness and impaired mitochondrial biogenesis dominate. EV-mediated miR-378 enhances PGC-1β/ERRγ-driven transcriptional programs, restoring ATP-generating capacity and fatty acid oxidation over extended periods, serving as a disease-modifying intervention.
3. Native vs. Engineered EVs: Native EVs reflect endogenous cardiomyocyte or progenitor signatures, offering baseline preventive or modulatory activity. Engineered EVs, in contrast, provide precision-tuned delivery, enabling organelle-specific targeting, miRNA enrichment, and temporally synchronized therapeutic action aligned with patient-specific bioenergetic thresholds [21,104,120].

This framework integrates disease stage, temporal responsiveness, and delivery modality, providing a mechanistically grounded roadmap for tailoring EV-encapsulated mito-miR therapy across diverse cardiac pathologies. It also emphasizes that mito-miRs function as dynamic, context-adaptive modulators of cardiomyocyte bioenergetics rather than uniform agents.

#### 3.5.2. Delivery Limitations and Contextual Challenges

Despite their promise, translational obstacles remain. Delivery inefficiency is a critical bottleneck, as a significant fraction of EVs fails to reach target cardiomyocytes or penetrate the mitochondrial compartment, reducing net bioenergetic impact [20]. Rapid clearance by the reticuloendothelial system or renal filtration further limits bioavailability, highlighting the need for temporal optimization of administration windows. Off-target uptake by non-cardiac cells may elicit unintended molecular rewiring, potentially perturbing systemic metabolic or redox homeostasis [66]. Notably, several preclinical studies report limited functional benefit despite successful EV uptake, reflecting insufficient miRNA copy number per vesicle, subthreshold mitochondrial engagement, or mistimed delivery outside the critical injury window. MicroRNAs are minor constituents of extracellular vesicles that are rarely delivered to target cells [121,122,123,124]

From a systems-level perspective, these limitations reflect the interplay of vesicle pharmacokinetics, tissue-specific tropism, and subcellular accessibility. Mitigation strategies—including surface ligand engineering, cardiomyocyte-directed tropism motifs, and adaptive dosing regimens—are actively explored, yet these challenges illustrate that EV-mediated mito-miR therapy is context-dependent and remains a finely balanced, network-sensitive intervention [25,111].

Native EVs offer favorable biocompatibility and immunological safety profiles, making them attractive for preventive or low-intensity metabolic modulation. In contrast, engineered EVs enable higher cargo stability, mitochondrial pathway specificity, and dosing precision, positioning them as more suitable for therapeutic intervention in established disease. These gains, however, are accompanied by increased complexity in manufacturing, biodistribution control, and long-term safety profiling, underscoring the trade-off between biological fidelity and therapeutic precision.

### 3.6. Intersections with Multi-Omics: scRNA-Seq, Epitranscriptomics, Proteomics

The therapeutic integration of EV-encapsulated mitochondrial miRNAs into cardiac disease paradigms increasingly depends on the ability to deconvolute molecular heterogeneity and dynamically model disease states at high resolution. Multi-omics technologies—particularly single-cell RNA sequencing (scRNA-seq), epitranscriptomics, and high-throughput proteomics—have transformed our understanding of cardiomyocyte subtypes, metabolic maturation stages, and spatially resolved pathology in the failing heart. These platforms are now instrumental not only in target identification but in evaluating the precision and off-target consequences of miRNA-based interventions.

Single-cell RNA-seq enables granular mapping of iPSC-CM differentiation trajectories, revealing transcriptional bottlenecks associated with metabolic immaturity, such as insufficient expression of mitochondrial transcription factors (e.g., TFAM, TFB2M) [2,3,4,5,6,7,8,9] and nuclear-encoded ETC components. When combined with spatial transcriptomics, these approaches delineate regional mitochondrial stress responses within infarcted or hypertrophic myocardium. Incorporating mito-miR perturbation studies into these platforms offers a mechanistic layer of resolution for understanding therapeutic rewiring at the single-cell level.

Epitranscriptomic modifications—particularly N6-methyladenosine (m^6^A) methylation—further shape miRNA biogenesis, stability, and EV packaging. Recent findings suggest that methylation-sensitive RBPs influence the selective loading of miRNAs into vesicles, while m^6^A-dependent processing modulates miRNA maturation kinetics within cardiomyocytes. These insights expand the scope of mitochondrial miRNA therapies, offering levers to control not just miRNA function but their pharmacodynamic behavior in vivo.

Complementary proteomic analyses of EV cargo and recipient cardiomyocyte responses illuminate downstream signaling cascades, particularly in redox-sensitive kinase networks (e.g., AMPK, MAPK, JNK). Moreover, phosphoproteomic signatures are beginning to be used to assess mitochondrial remodeling efficiency post-miRNA delivery, establishing feedback systems for therapeutic optimization.

Crucially, the integration of these omic layers is enabling the construction of computationally tractable models of cardiac metabolism and mitochondrial signaling. Machine learning and AI frameworks are now employed to predict miRNA–target interactions, model EV biodistribution, and simulate transcriptomic shifts under disease-specific stressors. These tools open unprecedented opportunities for in silico screening of mito-miR candidates, stratification of patient-specific response profiles, and dynamic iteration of EV engineering strategies in silico before translation into vivo platforms.

As these technologies converge, they create a scaffold for next-generation RNA therapeutics that are not only modular and adaptable, but also embedded in rational systems-level design. Within this expanding therapeutic frontier, mitochondrial miRNAs encapsulated in EVs represent a uniquely positioned modality that intersects seamlessly with emerging trends in mRNA-based interventions, antisense oligonucleotide therapies, and cell-free synthetic biology—directions explored in the following section.

### 3.7. Synergies with Cell-Free and RNA-Based Therapies

The advent of RNA-based therapies has fundamentally redefined the landscape of precision medicine. Within this evolving paradigm, extracellular vesicle (EV)-delivered mitochondrial miRNAs represent a compelling, cell-free complement to current nucleic acid platforms. Their inherent modularity, low immunogenicity, and endogenous biogenesis render them uniquely suitable for integration into next-generation RNA therapeutic strategies, including mRNA delivery systems, antisense oligonucleotide (ASO) platforms, and RNA-based gene modulation constructs.

Messenger RNA (mRNA) therapies, as exemplified by recent success in vaccine development, have underscored the translational viability of lipid-based RNA delivery. However, therapeutic mRNA applications targeting metabolic and mitochondrial dysfunction in cardiomyocytes remain in early stages due to challenges in stability, expression timing, and intracellular routing. EVs, by virtue of their native membrane structure and organelle-homing capacity, offer an elegant, biocompatible alternative for targeted delivery of regulatory RNAs that modulate the same mitochondrial pathways as mRNA-induced protein translation—achieving gene expression modulation without reliance on de novo protein synthesis.

Antisense approaches, including anti-miRs and locked nucleic acid (LNA) technologies, provide complementary strategies to inhibit pathogenic or maladaptive miRNA activity, including miRNAs that suppress mitochondrial maturation or promote fibrosis. The co-application of EVs to deliver synthetic anti-miRs alongside mitochondrial-targeting miRNAs enables dual-axis regulation: enhancing bioenergetic reprogramming while silencing deleterious transcriptional feedback loops. Such multiplexed delivery strategies have already shown promise in cardiac fibrosis models and are readily extendable to ischemic and metabolic heart diseases.

The modularity of EV systems also aligns closely with synthetic biology’s broader ambitions to develop programmable, smart therapeutics. EVs can be engineered to respond to stimuli such as pH, oxidative stress, or metabolite concentration, activating their cargo only in pathological microenvironments. This functional sophistication mirrors and complements the responsiveness being built into synthetic RNA switches and self-amplifying RNA systems—signaling a convergence point between biologically derived and synthetically designed delivery architectures.

The translational advantage of these convergences lies in their shared commitment to cell-free, tunable, and scalable therapeutics. Yet, despite their promise, EV-based mitochondrial therapies must still overcome several biological and engineering hurdles before reaching clinical deployment. Reproducibility of EV content, targeted delivery efficiency, immunological safety, and large-scale manufacturing remain areas of active investigation—issues explored in the following section.

While extracellular vesicle (EV)-encapsulated mitochondrial miRNAs offer a transformative approach to cardiac metabolic modulation, the pathway from conceptual innovation to clinical implementation remains encumbered by critical translational challenges. These challenges are not solely technical, but intersect with biological variability, immunological complexity, and bioengineering constraints, all of which must be addressed to achieve reproducibility, safety, and scalability.

### 3.8. Multi-Omics Integration of EV–miRNA-Mediated Mitochondrial Reprogramming

Integration of transcriptomic, proteomic, and metabolomic datasets provides a systems-level framework for understanding how EV-delivered mitochondrial microRNAs orchestrate cardiomyocyte bioenergetic remodeling. Transcriptomic analyses following EV–miRNA treatment consistently demonstrate coordinated upregulation of nuclear-encoded mitochondrial regulators, including genes governing OXPHOS, FAO, and mitochondrial dynamics, while concurrently suppressing stress-associated and apoptotic programs. These transcriptional shifts are not isolated events; rather, they propagate through the proteome, where increased abundance and stabilization of respiratory chain subunits, mitochondrial chaperones, and fusion-associated proteins reflect successful translation of upstream regulatory signals into functional mitochondrial architecture. Proteomic data further reveal improved stoichiometric balance across electron transport chain complexes, a feature strongly associated with restored respiratory efficiency and reduced electron leak.

Metabolomic profiling adds a critical functional layer to this framework by capturing downstream consequences of these molecular changes on cellular energy flux. EV–miRNA-treated cardiomyocytes exhibit increased tricarboxylic acid cycle intermediates, enhanced fatty acid-derived acetyl-CoA availability, and reduced accumulation of glycolytic byproducts, collectively indicating a shift toward oxidative, adult-like metabolic states. Importantly, reductions in oxidative stress markers and normalization of redox couples corroborate transcriptomic and proteomic evidence of improved mitochondrial integrity. When integrated, these multi-omics modalities converge on a unified model in which EV-delivered mito-miRs act as upstream regulators that rewire transcriptional programs, stabilize mitochondrial protein networks, and ultimately reshape metabolic flux toward efficient ATP generation. This convergence underscores the value of multi-omics pipelines—not as parallel descriptive tools, but as mutually reinforcing layers that together define bioenergetic restoration as an emergent, system-level outcome of EV-mediated miRNA delivery.

Together, these integrated omics layers reveal mitochondrial rescue not as a single molecular event, but as a coordinated systems response to EV-mediated miRNA delivery.

## 4. Challenges in EV-Based Mitochondrial Therapies

Reproducibility remains a fundamental concern in EV-based platforms, particularly due to batch-to-batch variation in vesicle content, size, and functional potency. Despite advances in vesicle isolation and purification—ranging from ultracentrifugation to microfluidic-based sorting—standardized protocols for ensuring consistent miRNA loading and vesicle homogeneity are still lacking. Moreover, cargo variability is influenced by donor cell state, culture conditions, and even subtle differences in exosomal biogenesis pathways, posing a barrier to both experimental reproducibility and regulatory approval.

These limitations mirror challenges described in iPSC-CM maturation platforms, where mitochondrial immaturity, batch variability, and incomplete bioenergetic maturation constrain translational fidelity. Recent integrative analyses emphasize that partial mitochondrial rescue—whether through metabolic conditioning, electromechanical stimulation, or EV-mediated mitochondrial transfer—remains insufficient to achieve adult-like mitochondrial competence unless coordinated with transcriptional and genomic control of mitochondrial biogenesis programs. This convergence highlights that EV-based mitochondrial therapies, when deployed in isolation, may be inherently limited by the same structural and regulatory bottlenecks observed in immature cardiomyocyte systems [13].

Importantly, several preclinical studies report incomplete or transient bioenergetic recovery following EV administration, with improvements in ATP content, mitochondrial membrane potential, or ROS buffering that fail to reach adult-like levels or dissipate over time [12,13,125]. In some models, EV delivery results in modest phenotypic benefit without durable mitochondrial network integration, highlighting that vesicle uptake alone does not guarantee functional mitochondrial rescue [126,127,128]. Negative or neutral outcomes are particularly evident when EV dosing, cargo stoichiometry, or recipient metabolic state are not tightly controlled, underscoring that EV-mediated delivery can fail to translate into meaningful bioenergetic restoration despite successful cellular internalization [129,130,131].

Targeted delivery is another pivotal limitation. While EVs possess natural tropism to injured tissues, their unmodified biodistribution remains suboptimal for precision mitochondrial delivery. Efforts to enhance targeting via surface engineering—such as mitochondrial localization sequences or cardiomyocyte-specific peptides—have shown promise but require further validation in large-animal and human models. Moreover, intracellular trafficking to the mitochondrial matrix remains inefficient and poorly characterized, highlighting the need for improved organelle-specific delivery systems.

Consistent with this, mitochondrial immaturity in iPSC-derived cardiomyocytes has been shown to reflect not only limited mitochondrial content but also impaired integration of mitochondrial networks, cristae organization, and calcium–energetic coupling—suggesting that effective mitochondrial targeting must extend beyond delivery to encompass functional network incorporation [121].

Immunogenicity is relatively low in native EV systems compared to synthetic nanoparticles or viral vectors. However, immune activation cannot be fully excluded, particularly in the context of repeated dosing, modified surface proteins, or xenogeneic donor sources. Comprehensive immunoprofiling of engineered EVs in diverse genetic backgrounds is essential to mitigate the risk of off-target immune responses and chronic inflammation.

Scalability represents perhaps the most pressing bottleneck. Current EV production platforms lack the yield, cost-efficiency, and GMP-compliant standardization required for widespread clinical use. Bioreactor-based systems, combined with synthetic biology approaches for inducible cargo expression and real-time cargo tracking, are under development but remain in early translational stages.

Addressing these multifaceted challenges will require a systems-level integration of nanotechnology, molecular biology, bioinformatics, and biomanufacturing disciplines. While these obstacles are substantial, they do not diminish the principled organizational sophistication of EV-mediated intercellular communication, which reflects conserved structural and functional motifs observed across complex biological networks. Furthermore, the architecture of vesicle-based signaling exhibits functional alignment with hierarchical regulatory frameworks, emphasizing coordinated information transfer and energetic optimization at the cellular and tissue level.

These platform-dependent limitations are systematically reflected in the comparative scalability metrics and manufacturing variability detailed in Table 3. The table also synthesizes functional recovery heterogeneity across delivery platforms, particularly with respect to bioenergetic restoration efficiency, including relative changes in ATP production, mitochondrial membrane potential (MMP), and reactive oxygen species (ROS) reduction. The superior performance of engineered EV platforms compared with native EVs, liposomes, and lipid nanoparticles is delineated therein, although organelle-level mitochondrial routing remains incompletely optimized. In addition, comparative immunogenicity profiles across delivery systems are summarized, providing a structured assessment of innate immune activation risk. Finally, the scalability gradient among native EVs, engineered EVs, liposomes, and lipid nanoparticles is clearly outlined, emphasizing translational feasibility constraints across platforms.

### Risks and Translational Constraints in EV-Mediated Mito-miR Delivery

Despite the therapeutic promise of EV-mediated mitochondrial microRNA delivery, several biological and translational risks warrant careful consideration to ensure both efficacy and safety. A primary concern is the potential for off-target mitochondrial effects arising from the pleiotropic nature of miRNA–mRNA interactions. While mito-miRs can enhance bioenergetic recovery in cardiomyocytes, excessive or ectopic modulation of mitochondrial gene networks in non-target tissues may disrupt oxidative balance, alter mitochondrial dynamics, or provoke maladaptive stress responses. This risk is particularly relevant for miRNAs such as miR-181c and miR-34a, whose overexpression has been linked to electron transport chain destabilization and accelerated mitochondrial aging, respectively. Precision EV surface engineering to achieve cardiomyocyte-restricted tropism, combined with controlled miRNA stoichiometry, is therefore essential to minimize unintended mitochondrial perturbations. The extensive overlap of mitochondrial miRNA expression across diverse disease states, as documented by Kaur et al. [16], reinforces the necessity of functional rather than diagnostic classification when designing bioenergetic interventions.

A second challenge concerns the intrinsic half-life and functional persistence of delivered miRNAs. Although miRNAs exhibit greater stability than many RNA species, their intracellular activity remains temporally constrained by degradation pathways, dilution during cell division, and dynamic competition for RNA-induced silencing complex (RISC) loading. In the setting of acute cardiac injury, insufficient duration of miRNA activity may fail to sustain mitochondrial transcriptional and metabolic programs beyond the initial rescue window. Conversely, prolonged or repeated exposure risks exceeding physiological regulatory thresholds, potentially perturbing tightly balanced bioenergetic or apoptotic signaling networks. These constraints underscore the necessity of aligning miRNA persistence with disease-specific temporal demands, potentially through EV engineering strategies that modulate cargo release kinetics or enable controlled, low-dose repeat administration.

Beyond intracellular durability, systemic clearance and biodistribution represent major determinants of therapeutic index. Circulating EVs are rapidly sequestered by the mononuclear phagocyte system, subjected to hepatic uptake, and eliminated through renal filtration, collectively limiting bioavailability within the myocardium. Accelerated clearance not only diminishes effective myocardial dosing but may also result in disproportionate accumulation of miRNA cargo in off-target organs such as the liver or spleen. Accordingly, advances in EV surface modification—including shielding of phagocytic recognition motifs or incorporation of cardiotropic ligands—are central to extending circulatory half-life while preserving tissue specificity. Such strategies are critical to balancing exposure, efficacy, and off-target risk.

Collectively, these observations indicate that EV-mediated mito-miR delivery, while biologically compelling, may yield variable or subtherapeutic outcomes in the absence of precise control over targeting fidelity, dosing kinetics, intracellular persistence, and systemic clearance [132,133,134]. Successful clinical translation will therefore depend not only on the intrinsic potency of mito-miRs, but on the rigorous integration of bioengineering strategies that harmonize delivery dynamics with mitochondrial and disease-specific regulatory constraints.

## 5. Future Directions or Conclusions

Mitochondrial function lies at the heart of cardiovascular health, and its disruption underpins some of the most intractable forms of heart disease. This review has proposed that extracellular vesicles (EVs), when precisely engineered to deliver mitochondrial microRNAs (mito-miRs), represent a biologically intelligent and clinically adaptable strategy for reprogramming cardiomyocyte energetics. At the intersection of RNA regulation, organelle biology, and nanotherapeutic innovation, EV-embedded mito-miRs emerge not as passive molecular additives, but as dynamic bioenergetic architects capable of restoring redox balance, enhancing OXPHOS, and activating transcriptional networks that resemble those of the mature myocardium.

Synthesizing evidence from stem cell biology, transcriptomics, and redox physiology, we have outlined how mito-miRs influence key regulatory nodes—from mitochondrial gene expression and ROS buffering to metabolic reprogramming and mitochondrial dynamics. When delivered via EVs, these small RNAs achieve spatiotemporally coordinated modulation of cardiomyocyte fate without invoking the risks associated with direct cell transplantation or synthetic transfection agents. In parallel, advancements in vesicle encapsulation, surface engineering, and computational modeling are making possible a new generation of targeted, programmable nanotherapies that operate with cellular-level precision and systemic-scale implications.

The therapeutic vision that emerges is one in which cardiovascular disease is no longer managed solely by symptomatic intervention, but reshaped at its molecular source through intelligent reconfiguration of metabolic identity. EV-based delivery of mitochondrial miRNAs embodies this paradigm shift—offering a platform that is biologically congruent, technologically versatile, and inherently scalable for translational application.

Ultimately, the concept of EV-embedded mitochondrial microRNAs positions RNA-based therapeutics as active regulators of cardiomyocyte bioenergetics rather than passive signaling agents. By modulating mitochondrial transcriptional programs, redox balance, and metabolic substrate utilization, EV-delivered mito-miRs offer a scalable strategy to restore energetic competence and functional resilience in injured myocardium. These vesicle-encapsulated miRNAs function as programmable molecular modulators capable of re-establishing mitochondrial homeostasis within defined quantitative and temporal thresholds, thereby supporting precision interventions aimed at preserving cardiomyocyte viability and contractile performance following cardiac injury.

## Figures and Tables

**Table 1:** Ranked canonical mitochondrial miRNAs and their bioenergetics targets.

Ranked	miRNA	Net ATP Gain	ROS Change	ETC Recovery/Stability	Primary Target Gene(s)	Dominant Biological Pathway	Impact on Cardiomyocyte Metabolism	Disease Context
1.	miR-378 [15]	↑↑↑	↓↓	High	PGC-1β, ERRγ, CPT1B	Mitochondrial biogenesis, FAO	Enhance oxidative metabolism and ATP production; promotes adult-like metabolic maturation	HFpEF, metabolic cardiomyopathy
2.	miR-499 [38,39]	↑↑	↓↓	High	SOX6, PDCD4	Mitochondrial dynamics, apoptosis resistance	Stabilizes mitochondrial networks; reduces apoptosis and supports contractile energy demands	Doxorubicin cardiotoxicity, HF
3.	miR-133a [32,35]	↑↑	↓↓	High	RhoA, Caspase-9	Mitochondrial survival signaling	Preserves mitochondrial function and ATP stability	Cardiomyopathy, HF
4.	miR-210 [40,41]	↔/↓	↓↓↓	Moderate	ISCU, COX10, SDHD	Hypoxia adaptation, ROS buffering	Suppresses mitochondrial respiration under hypoxia, preserves membrane potential and limits oxidative stress	IHD, myocardial ischemia
5.	miR-27b [42,43]	↑↑	↓	Moderate-High	PPARγ, CPT1A	Lipid metabolism, mitochondrial substrate utilization	Shifts fuel preference toward oxidative metabolism	Metabolic syndrome-associated HF
6.	miR-21 [44]	↑	↓↓	Moderate	PTEN, PDCD4	Stress signaling, mitochondrial survival	Indirectly enhances mitochondrial resilience under stress	Ischemic injury, myocarditis
7.	miR-30 family [32,36]	↑	↓	Moderate	BNIP3, Beclin-1	Mitophagy regulation	Controls mitochondrial turnover and quality control	HF, ischemic remodeling
8.	miR-146a [33,45]	↔/↑	↓↓	Moderate	TRAF6, IRAK1	Immunometabolic regulation	Limits inflammation-driven mitochondrial damage	Viral myocarditis, inflammatory cardiomyopathy
9.	miR-24 [46,47,48,49]	↑/↔	↓	Low-Moderate	BIM, PGC-1α (context-dependent)	Apoptosis–metabolism balance	Modulates mitochondrial survival pathways under oxidative stress	IHD, HF
10.	miR-92a [49,50]	↑	↓	Low-Moderate	KLF4, eNOS (indirect metabolic effects)	Endothelial–cardiomyocyte metabolic coupling	Alters metabolic substrate delivery and mitochondrial efficiency	IHD, microvascular dysfunction
11.	miR-23a/b [51,52]	↓	↑	Low	PGC-1α, MnSOD (Indirect)	Redox balance, mitochondrial stress response	Modulates ROS handling and mitochondrial antioxidant capacity	Chemotherapy-induced cardiomyopathy
12.	miR-181c [53,54]	↓/↔	↑	Low	MT-CO1 (COX 1), BCL2	OXPHOS regulation, apoptosis signaling	Modulates electron transport chain efficiency; excessive expression may impair complex IV activity and increase ROS	IHD, HF, ischemia–reperfusion injury
13.	miR-15 family (miR-15a/b) [55,56,57]	↓↓	↑	Impaired	ARL2, BCL2	Mitophagy, mitochondrial integrity	Excess activity impairs mitochondrial maintenance and ATP generation	Post-ischemic remodeling
14.	miR-195 [51,58,59]	↓↓	↑	Impaired	MFN2, BCL2	Mitochondrial fusion, apoptosis	Drives mitochondrial fragmentation and reduced respiratory efficiency	Pathological hypertrophy, HF
15.	miR-34a [60,61]	↓↓↓	↑	Severely Impaired	SIRT1, PNUTS	Mitochondrial aging, DNA damage response	Promotes mitochondrial dysfunction and energetic decline when upregulated	Aging heart, HFpEF

Arrow definitions are as follows: ↑ minimal increase (≤10–20% vs. control); ↑↑ moderate increase (~20–50%); ↑↑↑ robust increase (>50%). ↓ minimal reduction (≤10–20%); ↓↓ moderate reduction (~20–50%); ↓↓↓ marked reduction (>50%). ↔ no significant change (<10% variation or context-dependent neutrality). “Impaired” denotes reproducible ETC destabilization, ΔΨm loss, or reduced complex activity across cardiomyocyte-relevant models. ATP gain refers to net intracellular ATP production; ROS change denotes pathological mitochondrial ROS (primarily superoxide/H_2_O_2_); ETC recovery reflects restoration of complex activity, cristae integrity, and ΔΨm stabilization relative to disease baseline.

**Table 2:** Disease-Specific EV Payload Design for Mitochondrial Bioenergetic Restoration.

Disease Context	Dominant Mitochondrial Defect	EV-Delivered miRNAs (Augment)	miRNAs to Inhibit (Anti-miR EVs)	Expected Bioenergetic Outcome
Ischemic Heart Disease (IHD)	Acute ETC disruption [68,69], ROS surge [70,71], ΔΨm collapse [72,73]	miR-210, miR-21, miR-133a	miR-34a, miR-15 family	Preserved membrane potential, reduced ROS, improved post-ischemic survival
Ischemia–Reperfusion Injury	Oxidative burst [74], mitochondrial permeability transition [75]	miR-210, miR-146a, miR-30 family	miR-34a, miR-195	ROS buffering, improved mitochondrial recovery, reduced apoptotic signaling
HFpEF (Metabolic/Aging-Driven)	Impaired FAO [76,77], reduced biogenesis [78], energetic stiffness [79]	miR-378, miR-27b, miR-133a	miR-34a, miR-23a/b	Enhanced oxidative metabolism, ATP restoration, adult-like metabolic profile
Chronic HFrEF	Mitochondrial network instability [80,81], ATP insufficiency [82]	miR-499, miR-378, miR-133a	miR-195, miR-15 family	Stabilized mitochondrial networks, sustained ATP output
Doxorubicin Cardiotoxicity	mtDNA damage [83,84], apoptosis-driven ETC loss [85]	miR-499, miR-21, miR-30 family	miR-23a/b, miR-34a	Preserved mitochondrial integrity, reduced cardiomyocyte loss
Viral Myocarditis	Inflammation-driven mitochondrial injury [86]	miR-146a, miR-21	miR-34a	Immunometabolic stabilization, protection from cytokine-induced dysfunction
Metabolic Syndrome-Associated HF	Fuel inflexibility [87], lipid overload [88]	miR-378, miR-27b	miR-23a/b	Improved substrate utilization, reduced oxidative stress
Post-Ischemic Remodeling	Defective mitophagy [89,90], maladaptive hypertrophy [91,92]	miR-30 family, miR-133a	miR-15 family, miR-195	Improved mitochondrial quality control and structural preservation

Disease-specific EV payload design enables mitochondrial repair strategies to be matched to distinct energetic failure modes, rather than applied uniformly across cardiac pathologies. Accordingly, individual miRNAs may appear across multiple disease contexts depending on whether classification is observational, mechanistic, or therapeutic.

**Table 3:** Translational Performance Matrix of EV-Based Mito-miR Therapy.

Delivery Platform	Mitochondrial Targeting Efficiency [1,2,3,4,5,6,7,8,9,10,11,12,13,17,92,93,94,95,96,97,100,101,102,103]	Immunogenicity [1,2,3,4,5,6,7,8,9,10,11,12,13,87,88,89,90]	Scalability [12,87,88,89,90]	Biocompatibility [12,13,17,92]	Bioenergetic Restoration(ATP ↑/ROS ↓/MMP ↑) * [8,9,18]
Native EVs [8,9,23,86]	Moderate Passive mitochondrial engagement via endogenous trafficking; limited organelle specificity	Very low, Immune-silent, self-derived lipid/protein signatures	Low–Moderate yield variability; donor-cell dependent	Very high physiologic membranes, minimal cytotoxicity	Moderate Improved mitochondrial resilience; partial ATP recovery
Engineered EVs [17,92,93,97,100,101,102]	High Enhanced via MTS/MLS peptides, Lamp2b fusions, or RNA-binding adaptors	Low Minimal innate immune activation if properly purified	Moderate scalable with standardized producer cell lines	High preserved membrane fusion and intracellular routing	High–Very High Robust ATP restoration, ROS suppression, MMP stabilization
Liposomes [21,60,92]	Low–Moderate Limited mitochondrial access without targeting moieties	Moderate Complement activation and uptake by phagocytes reported	High Well-established industrial manufacturing	Moderate Artificial lipid composition; dose-dependent toxicity	Low–Moderate Indirect metabolic effects; inconsistent mitochondrial rescue
Lipid Nanoparticles (LNPs) [14,15,56]	Low Primarily cytosolic delivery; minimal mitochondrial routing	Moderate–High Innate immune sensing (TLR, interferon pathways)	Very high Clinically proven large-scale production	Moderate Transient inflammation limits repeat dosing	Low Effective gene silencing but limited direct bioenergetic recovery

* ↑ represents increase, ↓ represents decrease.

## Data Availability

No new data were created in this study. Data sharing is not applicable to this article.

## Abbreviations

AMPK	AMP-activated protein kinase
ASO	Antisense oligonucleotide
ATP	Adenosine triphosphate
BNIP3	BCL2 interacting protein 3
CPT1A/CPT1B	Carnitine palmitoyltransferase 1A/1B
CRISPR	Clustered regularly interspaced short palindromic repeats
COX1/COX10	Cytochrome c oxidase subunit I/assembly factor 10
ΔΨm	Mitochondrial membrane potential
DNA/mtDNA	Deoxyribonucleic acid/mitochondrial DNA
ECM	Extracellular matrix
ERRγ	Estrogen-related receptor gamma
ESCRT	Endosomal sorting complexes required for transport
ETC	Electron transport chain
EV(s)	Extracellular vesicle(s)
FAO	Fatty acid oxidation
GGAG	Glycine–glycine–alanine–glycine RNA motif
HF	Heart failure
HFpEF	Heart failure with preserved ejection fraction
HFrEF	Heart failure with reduced ejection fraction
hnRNPA2B1	Heterogeneous nuclear ribonucleoprotein A2/B1
IHD	Ischemic heart disease
iPSC-CM(s)	Induced pluripotent stem cell-derived cardiomyocyte(s)
IRAK1	Interleukin-1 receptor-associated kinase 1
ISCU	Iron–sulfur cluster assembly enzyme
JNK	c-Jun N-terminal kinase
Lamp2b	Lysosome-associated membrane protein 2B
LNA	Locked nucleic acid
MAPK	Mitogen-activated protein kinase
MFN2	Mitofusin 2
miRNA/miR	MicroRNA
mito-miR(s)	Mitochondrial and mitochondria-associated microRNA(s)
MnSOD	Manganese superoxide dismutase
MPI	Mito-miR Potency/Fidelity Index
MSC	Mesenchymal stem cell
MTS/MLS	Mitochondrial targeting sequence/mitochondrial localization signal
NF-κB	Nuclear factor kappa B
NRF2	Nuclear factor erythroid 2-related factor 2
OXPHOS	Oxidative phosphorylation
PDCD4	Programmed cell death protein 4
PGC-1α/PGC-1β	Peroxisome proliferator-activated receptor gamma coactivator 1 alpha/beta
PI3K	Phosphoinositide 3-kinase
PPARα/PPARγ	Peroxisome proliferator-activated receptor alpha/gamma
RBP	RNA-binding protein
ROS	Reactive oxygen species
scRNA-seq	Single-cell RNA sequencing
SDHD	Succinate dehydrogenase complex subunit D
SYNCRIP	Synaptotagmin-binding cytoplasmic RNA-interacting protein
TFAM	Mitochondrial transcription factor A
TFB2M	Mitochondrial transcription factor B2
TRAF6	TNF receptor-associated factor 6
YBX1	Y-box binding protein 1
